# Microplastics in Sea Turtles, Marine Mammals and Humans: A One Environmental Health Perspective

**DOI:** 10.3389/fenvs.2020.575614

**Published:** 2021-02-16

**Authors:** Idoia Meaza, Jennifer H Toyoda, John Pierce Wise

**Affiliations:** Wise Laboratory of Environmental and Genetic Toxicology, Department of Pharmacology and Toxicology, University of Louisville, Louisville, KY, United States

**Keywords:** microplastics, marine mammals, sea turtles, human, one health

## Abstract

Microplastics are ubiquitous pollutants in the marine environment and a health concern. They are generated directly for commercial purposes or indirectly from the breakdown of larger plastics. Examining a toxicological profile for microplastics is a challenge due to their large variety of physico-chemical properties and toxicological behavior. In addition to their concentration, other parameters such as polymer type, size, shape and color are important to consider in their potential toxicity. Microplastics can adsorb pollutants such as polycyclic aromatic hydrocarbons (PAHs) or metals on their surface and are likely to contain plastic additives that add to their toxicity. The observations of microplastics in seafood increased concern for potential human exposure. Since literature considering microplastics in humans is scarce, using a One Environmental Health approach can help better inform about potential human exposures. Marine mammals and sea turtles are long-lived sentinel species regularly used for biomonitoring the health status of the ocean and share trophic chain and habitat with humans. This review considers the available research regarding microplastic and plastic fiber exposures in humans, marine mammals and turtles. Overall, across the literature, the concentration of microplastics, size, color, shape and polymer types found in GI tract and feces from sea turtles, marine mammals and humans are similar, showing that they might be exposed to the same microplastics profile. Additionally, even if ingestion is a major route of exposure due to contaminated food and water, dermal and inhalation studies in humans have provided data showing that these exposures are also health concerns and more effort on these routes of exposures is needed. *In vitro* studies looked at a variety of endpoints showing that microplastics can induce immune response, oxidative stress, cytotoxicity, alter membrane integrity and cause differential expression of genes. However, these studies only considered three polymer types and short-term exposures, whereas, due to physiological relevance, prolonged exposures might be more informative.

## Graphical abstract

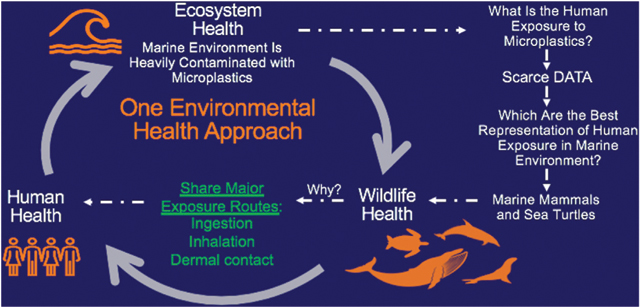

## INTRODUCTION

Plastics are typically composed of a variety of polymers and additives used to impart unique properties, such as lightweight, thermal and electrical insulation, durability, corrosion-resistance, and tensile strength (Andrady and Neal, 2009). The usefulness and low cost of these materials for diverse applications is responsible for the increase in worldwide plastics production from two million tonnes in 1950 (Geyer et al., 2017) to 359 million tonnes in 2018 (PlasticEurope, 2019). Microplastics originate from breakdown of larger plastics or are specially manufactured for use in products, such as toothpaste or skincare products. Microplastics are defined as “any synthetic solid particle or polymeric matrix, with regular or irregular shape and with size ranging from 1 um to 5 mm” (Frias and Nash, 2018). Their small size and durability have allowed them to become ubiquitous. Physical and chemical properties of microplastics could determine their toxicity (Wright and Kelly, 2017). Physical properties including size, shape and particle density influence the transport and fate of particles (Chubarenko et al., 2016). Chemical composition, including manufacturing materials such as polymers, colorizers (e.g., chromium), UV stabilizers (e.g., lead and cadmium) and flame retardants (e.g., aluminum oxide), as well as contaminants from the environment that attach to the surface through sorption (e.g., metals and persistent organic pollutants) can include hazardous compounds (Campanale et al., 2020).

The marine environment is a major sink for microplastics. These microplastic particles can enter the ocean through a variety of land and sea sources and have been found from the sea surface all the way to the seafloor and along the shoreline (GESAMP 2016; FAO 2018). River runoff is considered one of the major sources of plastic pollution in seawater. Indeed, Lebreton et al. (2017) estimated 67% of the plastic pollution in the ocean started in twenty rivers, mainly located in Asia. Another important source contributing to marine microplastic pollution is the widespread use of plastics in fisheries and aquaculture such as disposable fishing gear, plastic cages, packages and buoys. Consequently, Lusher et al. (2017) found that over 220 species of marine animals (excluding birds, turtles and mammals) ingested microplastic, of which half of them are considered relevant for commercial purposes and increase the risk of human consumption of microplastics.

In particular, large marine vertebrates, such as marine mammals and sea turtles, are key species for microplastic biomonitoring (Galgani et al., 2014). Exposure to environmental microplastics can occur though ingestion, inhalation and dermal contact though most research so far has focused on inhalation and ingestion (Revel et al., 2018, Prata et al., 2020). Marine mammals and sea turtles integrate all three exposure routes, a feature they share with humans, which makes them more representative of human exposures in the marine environment. Ingestion of these particles has received the most attention due to the presence of microplastics in commonly used products, such as sugar (0.44 microplastic/g), honey (0.1 microplastic/g), salt (0.11 microplastic/g), alcohol (32.27 microplastics/l), bottled water (94.37 microplastic/g), tap water (4.23 microplastic/l) and seafood (1.48 microplastic/g) (Cox et al., 2019). The presence of microplastics in seafood raises concern about potential bioaccumulation and biomagnification of microplastics in the trophic chain (van Raamsdonk et al., 2020). Marine mammals and sea turtles are likely to ingest similar microplastics as humans because they share similar marine trophic chains, and therefore can reveal valuable information on trophic transfer of microplastics (Carbery et al., 2018). One might argue that humans have a more diverse diet that may include things like alcohol or beverages that contain microplastics (Cox et al., 2019); however, marine mammals and sea turtles, are the best animal representation of humans in the marine environment, which is the major sink of microplastics and thus, important insights may still be gleaned from these comparisons.

Marine mammals, sea turtles and humans are all air breathers, which makes them susceptible for particle inhalation. The presence of microplastics in air have been extensively studied in the past years (Zhang et al., 2020). These studies show that atmospheric deposition transports microplastic particles to the ocean surface air (Liu et al., 2019; Wang et al., 2020; Szewc et al., 2021), therefore, making marine air breathers highly susceptible to microplastic inhalation. Currently, there is no literature on microplastic inhalation, however it is clear they do inhale airborne particles as several studies reported the inhalation of HgSe particles in *Tursiops truncatus* and *Globicephala macrohynchus* (Rawson et al., 1995) and presence of accumulation of macrophages loaded with fine carbon particles in *Tursiops truncatus* resulting in anthracosis (Rawson et al., 1991), which is commonly reported in human autopsies, suggesting that the inhalation exposure of air-breathers is similar. Additionally, marine mammals and sea turtles are extremely vulnerable to inhaling airborne microplastics because they rapidly exchange big masses of air before diving and hold their breath during prolonged dives, resulting in a larger magnitude and exposure of the inhaled contaminant (Takeshita et al., 2017). Furthermore, marine mammals lack nasal turbinate structures responsible for filtering the air and trapping particles, enabling them to sneeze out the particles (Takeshita et al., 2017). Similarly, sea turtles lack turbinate structure except for *Dermochelys*, which is an exemption within reptiles (Davenport et al., 2009). Yet, despite their vulnerability, inhalation of airborne contaminants by marine mammals and sea turtles is often overlooked. Given the health concerns about marine microplastics and the importance of these sentinel species, this review considers the available research regarding microplastic and plastic fiber exposures in humans, marine mammals and turtles.

## METHODS

### Search Strategy

International databases including PubMed and ScienceDirect were searched for articles published up to the date of search. First search was carried out on 3/23/2020 for marine mammals and human related articles in PubMed and ScienceDirect databases. Searches included: 1) ((microplastics) and human) and epidemiology, 2) ((plastic fibers) and human) and epidemiology 3) (microplastics) and (the word of interest) 4) (plastic fibers) and (word of interest). The words of interest were the following: *Pinnipedia*, pinnipeds, *Otariidae*, sea lion, fur seal, *Phocidae*, true seal, seal, *Odobenidae*, walrus, *Mustelidae*, sea otter, *Ursidae*, polar bear, *Cetacea*, *Odontoceti*, *Physeteridae*, sperm whale, *Kogiidae*, pigmy sperm whale, dwarf sperm whale, *Ziphiidae*, beaked whale, *Platanistidae*, south Asian river dolphin, Ganges river dolphin, bhulan, Iniidae, amazon river dolphin, Bolivian bufeo, common boto, *Lipotidae*, Yangtze river dolphin, *Pontoporiidae*, franciscana, toninha, *Monodontidae*, beluga, narwhal, *Delphinidae*, dolphin, killer whale, pilot whale, grampus, Tucuxi, *Phocoenidae*, porpoise, *Mysticeti*, *Neobalaenidae*, pigmy right whale, *Balaenidae*, bowhead whale, North Atlantic right whale, North Pacific right whale, *Eschrichtiidae*, gray whale, *Balaenopteridae*, rorqual, *Sineria*, *Trichechidae*, manatee, *Dugongidae*, dugong, whale, marine mammals and human. Additionally, all the words of interest were searched for plural and singular forms when possible in order to avoid missing papers.

The literature searches for sea turtles were performed on April 15, 2020. PubMed and ScienceDirect databases were explored for searches including (Microplastics) AND (the word of interest) and (plastic fibers) AND (word of interest). The words of interest were the following: *Cheloniidae*, *Dermochelyidae*, green sea turtle, *Chelonia mydas mydas*, *Chelonia*, *mydas agassizii*, loggerhead sea turtle, *Caretta caretta*, Kempś ridley sea turtle, *Lepidochelys kempii*, Olive ridley sea turtle, *Lepidochelys olivacea*, hawksbill sea turtle, *Eretmochelys imbricate*, flatback sea turtle, *Natator depressor*, Leatherback sea turtle, *Dermochelys coriacea* and sea turtle. Additionally, all the words of interest were searched for plural and singular forms when possible in order to avoid missing papers.

We further considered relevant articles found referenced by the articles under consideration in the review.

### Exclusion Criteria

From all the results obtained we excluded: 1) Articles not related to the review topic, 2) Review articles, 3) Articles not containing primary data, such as articles based on prediction models. Due to the uniqueness of marine mammal samples, variation in methodology and data reporting criteria was to be expected and, therefore, no further exclusion criteria were applied.

## RESULTS

### Microplastics in Sea Turtles

Five studies considered microplastics in sea turtles (Table 1). Microplastics were found in all seven sea turtle species: green sea turtle (*Chelonia mydas*), loggerhead sea turtle (*Caretta caretta*), Kemp’s ridley sea turtle (*Lepidochelys kempii*), olive ridley sea turtle (*Lepidochelys olivacea*), hawksbill sea turtle (*Eretmochelys imbricate*), flatback sea turtle (*Natator depressus*), and leatherback sea turtle *(Dermochelys coriacea*) (Table 1). Each study focused on characterizing microplastic particles in the GI tract with four studies characterizing environmental levels of microparticles found in 144 wild sea turtles from 7 species, while the fifth administered microplastic particles to study gut passage time. The data are insufficient to consider any species-specific patterns.

**FIGURE 1 |:**
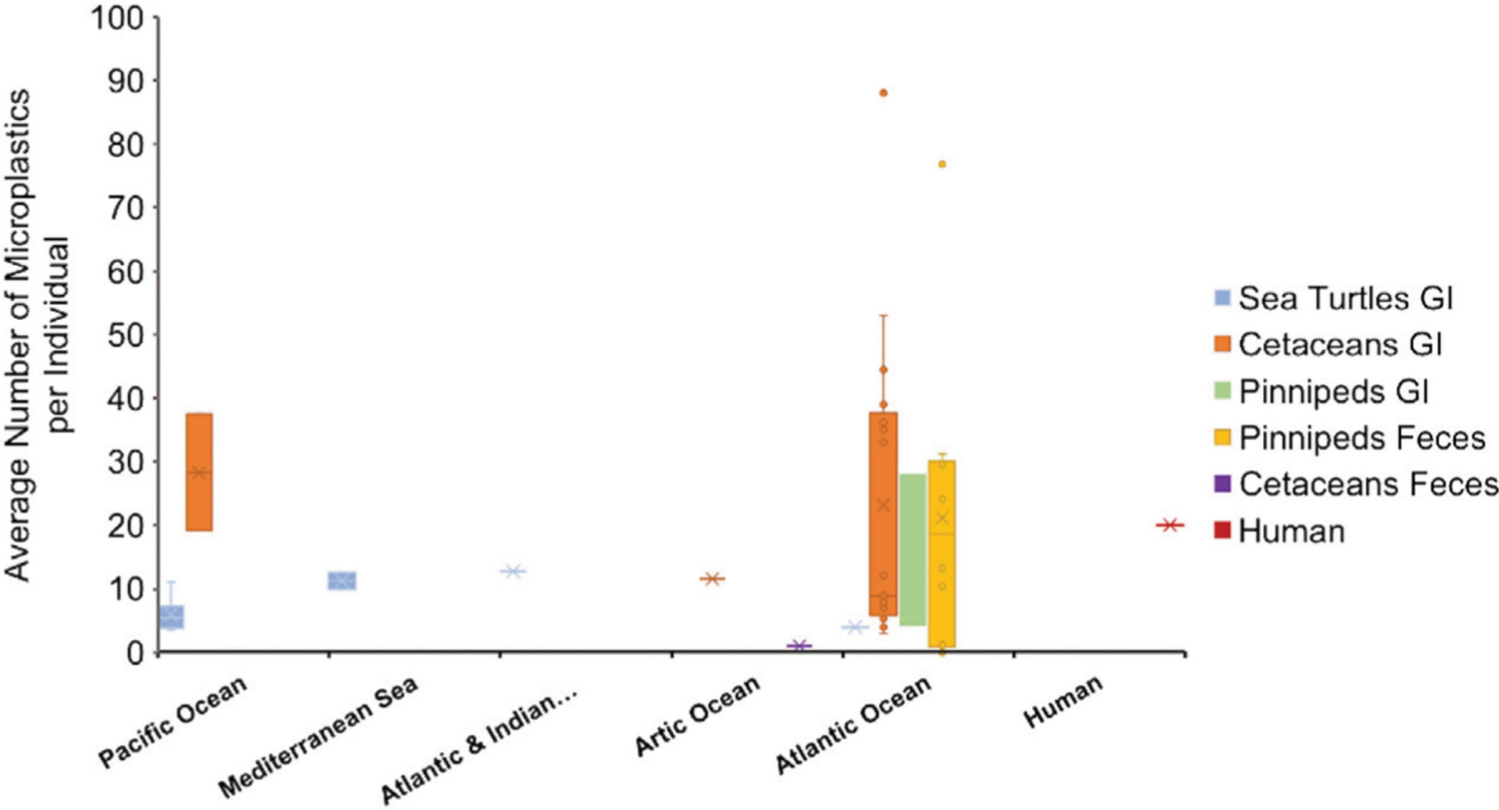
Average number of microplastics per individual based on animal groups and location, represented in a boxplot-chart. Data were extracted from Tables 1–4, representing the average number of microplastics per individual in each species. When the number of microplastics was given in the original article by shape (for example, number of pellets per individual and number of fragments per individual) the numbers were summed (Ryan et al., 2016; Donohue et al., 2019; Perez-Venegas et al., 2020). Only the information from the adult individuals in Zhu et al. (2019) were considered. Articles only reporting a range were not plotted on the boxplot (Nelms et al., 2018; Perez-Venegas et al., 2018). Human data (value of 20 microplastics/individual) represent the median value reported by Schwabl et al. (2019), with a range of 18–172 microplastics per individual.

The four studies of environmental levels of gut microparticles each documented microplastic particles in the digestive contents of the gut and characterized the physico-chemical aspects of the particles (Table 1). Particle size ranged from 0.1 to 5 mm, with mean sizes ranging from 1.4–4.7 mm, depending on the study. The average particle concentrations from the GI tract in sea turtles ranged from 2.5 to 12.5 particles per turtle (Figure 1). Particle shapes were predominately fibers and fragments and the most prevalent colors were blue, black, clear and white. Three studies (Pham et al., 2017; Caron et al., 2018; Duncan et al., 2019) reported polymer composition with polyethylene, ethylene propylene, polypropylene, polyester, polyacrylamide, polystyrene, polyamide, cellulose and elastomers most frequently found. One study (Duncan et al., 2019) compared different polymer types and turtles found in three different bodies of water but found no correlation between polymer type and the location of the turtles.

The fifth study (Amorocho and Reina, 2008) administered microplastic beads to wild juvenile East Pacific green turtles (*Chelonia mydas agassizii*) kept in captivity during the experiment for over 30 days and measured passage time of the microparticles. They administered different diets to the turtles together with microplastic beads and measured recovery of the beads. Cylindrical yellow beads of 2–3 × 1 mm size were packaged at a concentration of 20 beads per capsule. 3–5 capsules were introduced into the sea turtle lower esophagus by pushing them through a plastic hose. The average ingesta passage time in 6 turtles was 23.3 ± 6.6 days (559 h). Turtles fed with protein-based diet seemed to have longer ingesta passage time than turtles fed with mixed or plant-based diets, showing that diet might affect the retention time in the gut and therefore exposing the turtles to more prolonged exposures. Unexpectedly, 12 days after the initiation of the experiment one turtle died due to a hook ingested prior to the study and its necropsy showed microplastic beads were localized within boluses distributed along the midgut (Amorocho and Reina, 2008). This outcome further suggests food interacts with the microplastic particles and diet likely alters ingesta passage time of the beads. However, this study does not investigate such interaction or the potential breakdown of the microplastics.

Passage time was also noted in Pharm et al. (2017). In this study the color of macroplastics and microplastics in the GI tract did not match in some turtles. While microplastics can arise from breakdown of macroplastics, in this case the localization of microplastics with unique colors suggests that either they were ingested in the microplastic form or that they have a longer passage time through the gut than their microplastic source. Additionally, Pharm et al. (2017) not only identified macro, meso and microplastic ingestion in loggerhead sea turtles, but also showed that microplastics were mostly localized in the intestine, compared to the esophagus and stomach, suggesting a longer retention time in the intestine.

### Microplastics in Marine Mammals

16 articles regarding marine mammals met our selection criteria. From those, 9 studies analyzed presence of microplastics in the GI content of 15 cetaceans and 2 pinniped species from Atlantic, Pacific and Arctic Oceans (Table 2). From those 15 cetacean species, only one was mysticetes (*Megaptera novaeangliae*) and 14 were odontocetes. Additionally, 7 articles analyzed microplastics from fecal samples of 8 pinniped species and one odontocete (Table 3).

The average number of microplastics found in the GI tracts of large odontocetes ranged from 9 to 88 microplastics/individual, small odontocetes ranged 3 to 45 microplastics/individual and the only mysticete analyzed contained 6 items (Figure 1, Table 2). Lusher et al. (2018) found microplastic quantities in small odontocetes from Ireland, such as *Delphinus delphis, Stenella coeruleoalba, Phocoena phocoena* and *Tursiops truncatus,* that are comparable to the levels observed in bigger odontocetes, such as *Ziphius cavirostris* and *Orcinus orca,* analyzed in the same study. High levels of microplastics in small cetaceans could be reflective of coastal behavior, which puts them at higher risk of plastic ingestion.

The average number of microplastics per individual in the GI tracts of two pinniped species (*Phoca vitulina* and *Halichoerus grypus)* ranged between 4 and 27.9 microplastics/individual (Figure 1, Table 2). These values are similar to the number found in feces of pinniped species. However, one study showed alarmingly high presence of microplastics in *Arctocephalus australis* with values ranging from 0 to up to 180 microfibers per scat (Perez Venegas et al., 2018) (Figure 1, Table 3). Additionally, half of scat sub-samples from grey seals living in a sanctuary contained microplastics, where anthropogenic contamination is low. This evidence shows the ubiquity of microplastics even in controlled or less polluted areas (Nelms et al., 2018).

The size range of the microplastics found in the GI contents and feces were highly heterogenous ranging from 0.1 to 5 mm. Fibers were the most abundant shape of microplastics (Lusher et al., 2015; Hernandez-Gonzalez et al., 2018; Lusher et al., 2018; Xiong et al., 2018; Nelms et al., 2019; Zhu et al., 2019) in the GI tract content (Table 2), whereas fragments were more ubiquitous in the feces from 5 out of the 7 selected studies (Erisksson and Burton, 2003; Nelms et al., 2018; Donohue et al., 2019; Hudak et al., 2019; Moore et al., 2020) (Table 3). Among the selected studies in this review, one study looking at GI tract content and another study looking at the feces were unable to measure fibers due to the lack of procedural blanks (Besseling et al., 2015; Hudak et al., 2019).

Color and polymer type are two additional characteristics often reported in articles regarding microplastics. Among all the colors found, blue, black, white/clear/transparent and green are the most commonly observed in GI tract contents (Hernandez-Gonzalez et al., 2018; Lusher et al., 2018; Xiong et al., 2018; Nelms et al., 2019; Zhu et al., 2019) (Table 2) and fecal samples (Table 3). Additionally, fecal samples often contained red, purple, brown, green and yellow (Table 3). The spectrum of polymers observed is heterogeneous in the GI tract and fecal samples. Most common polymers were ethylene propylene, polypropylene, polyethylene, polyester, cotton, nylon and polyether sulfone (Tables 2,3). However, others such as polystyrene, polycarbonate, cellulose, polyolefin, polyvinyl chloride, acrylic, polyamide resin, low density polyethylene, poly(ethylene:prolypene:diene) rubber, alkyd resin and cellophane have been also found (Tables 2, 3). Most studies that analyzed the polymer type used FTIR (Tables 2, 3) and only one used Raman spectroscopy (Xiong et al., 2018) (Table 3). However, in some cases authors only analyzed a subsample of the fragments and fibers, due to the large quantity (Lusher et al., 2015; Donohue, 2019; Nelms, 2019; Perez Venegas, 2020).

Procedural blanks are extremely important to control for contamination during isolation of microplastics, especially fibers, since they are ubiquitous in the laboratory. From Table 2, 6 studies out of 9 (Lusher et al., 2015; Xiong et al., 2018; Hernandez-Millan et al., 2019; Nelms et al., 2019; Zhu et al., 2019; Moore et al., 2020) and in Table 3, only 3 studies mentioned specifically using procedural blanks to control for contamination (Nelms et al., 2018; Perez-Venegas et al., 2018; Donohue et al., 2019). The size, shape, color and polymer type of microplastics isolated from GI tract content is highly limited by the methods used during the isolation and characterization process.

Non-dietary ingestion of microplastics might account for a high percentage of total ingestion. Hernandez Millan et al. (2019) analyzed microplastics in grey seals and estimated theoretical ingestion of microplastics using the estimation by Lusher et al. (2013) of 1.9 microplastics ingestion per fish consumed. According to their results, 67% of the total amount of particles they observed were from dietary origin, therefore suggesting non-dietary ingestion occurs. Interestingly, Xiong et al. (2018) observed presence of microplastics in neonatal porpoise at levels that were comparable to the adults, suggesting a high rate of non-dietary ingestion. Moreover, Zhu et al. (2019) found microplastics in a newborn calf of a coastal delphinid species (*Sousa chinensis*), although at lower quantities.

Multiple studies analyzed the concentration of microplastics across different sections of the GI tract. Nelms et al. (2019) showed higher concentrations of microplastics in stomachs compared to intestines and Lusher et al. (2018) showed no correlation between number of microplastics and section of GI tract. In both studies, the majority of the microplastics were fibers. Lusher et al. (2015) showed that out of 88 particles isolated from a *Mesoplodon mirus* individual, 29 were located in the stomachs and 59 in the intestines, of which 89% were fibers. Xiong et al. (2018) showed similar results, a retention of fibers in the first sections of the intestine.

### Microplastics in Humans

22 articles regarding human exposure to microplastics were further reviewed. From those, 7 papers were based on *in vitro* studies using human cells, two used artificial digestion to understand the effects of human digestive fluids on the microplastics, 3 analyzed microplastics in human samples and 10 studied the effect of different toxicants involved in plastic manufacturing and associated risk of developing pathologies.

#### Microplastics in Human Samples: Lung and Feces

Three articles analyzed microplastics in human samples and all of them show presence of microplastics in human body (Pauly, 1998; Schwabl et al., 2019; Yan et al., 2020) (Table 4). Each of the studies had rigorous procedural blanks showing lack of contamination throughout the analysis. Two studies out of three investigated the presence of microplastics in human feces (Schwabl et al., 2019; Yan et al., 2019). Schwabl et al. (2019) and Yan et al. (2020) showed microplastics present in 100% and 40% of the fecal samples respectively. Schwabl et al. (2020) reported a median of 20 microplastics per 10 g of stool, with a range of 18–172 per 10 g of stool (Figure 1, Table 4). From those microplastics most were fragments or films with a size range of 50 um to 500 um. This study observed 9 polymer types by FTIR (polyethylene, polyester, polypropylene, polystyrene, polyamide (nylon), polyvinyl chloride, polyoxymethylene, polycarbonate, polyurethane) although polypropylene and polyester were most ubiquitous. On the contrary, Yan et al. (2020) identified polybutylene terephthalate and polyvinyl ether by Rama spectroscopy. Yan et al. (2020) did not provide information on size range or shape of microplastics found.

The third study, Pauly et al. (1998), for the first time identified patient tissue samples of nonneoplastic lung and malignant lung specimens contained inhaled plastic fibers. The authors suggest that fibers might increase the risk of developing lung disease.

#### Artificial Digestion System

Stock et al. (2020) and Liao and Yang (2020) studied the changes in microplastics during artificial digestions by whole digestive system *in vitro* method (WDSM). Both papers used synthetic gastric juices with different pHs and included shaking steps to mimic digestion steps. Saliva juices were shaken for 5 min, gastric juices for 1 h (Liao and Yang 2020) or 2 h (Stock et al., 2020). Intestinal juice was then shaken for 2 h (Stock et al., 2020) or4 h (Liao and Yang, 2020), and finally Liao and Yang (2020) added an additional step of large intestinal phase of 18 h.

Stock et al. (2020) investigated polyethylene, polyester, polyvinyl chloride, polypropylene and polystyrene microplastic polymers and observed that polystyrene particles showed changes in size and shape. These particles developed an irregular surface after the digestive steps and diameters increased up to 20 um through the different digestion steps. The rest of the polymer types were less affected by the digestive processes.

Interestingly, Liao and Yang (2020) analyzed polyester, polyvinyl chloride, polypropylene, polystyrene and polylactic acid polymers loaded with chromium (Cr), which simulates the release of toxicants that are attached to the microplastics throughout digestion. Oral bioaccessibility of Cr(VI) and Cr(III) was negligible in the mouth phase. However, the bioaccessibility of Cr(VI) in the gastric phase was significantly higher than those in the intestinal phases (small and large). For Cr(III) the highest bioaccessibility was on the small intestine. However, the levels were smaller to those found for Cr(VI) in gastric phase. Comparing between microplastic types, polylactic acid showed a higher release of Cr(VI) in each digestive phase.

#### *In vitro* Studies in Human Cells

*In vitro* studies looked at a variety of endpoints: cell viability, intracellular localization, oxidative stress, membrane integrity and immune response are summarized in Table 5 and Table 6. Overall, these *in vitro* studies assessed exposure to polystyrene, polyethylene and polypropylene polymer type microplastics.

Cell viability was reduced after exposure to microplastics in 4 studies (Helser et al., 2019; Hwang et al., 2019; Stock et al., 2019; Dong et al., 2020), but was not affected in other 3 studies (Schirinzi et al., 2017; Wu et al., 2019; Wu et al., 2020) (Table 5). No effects were found when cells were exposed to polystyrene for 12 h (0.1 or 5 um), 24 h or 48 h (5 um) and 24 h (10 um) or to polyethylene for 24 h (3–16 um). However, smaller particles of polystyrene (1, 1.72 or 4 um) induced a reduction in cell viability after 24 h, which was further decreased after prolonged (48 h) exposure to human epithelial colorectal adenocarcinoma cells (Caco-2) and human lung bronchial epithelial (BEAS-2B) cells (Stock et al., 2019; Dong et al., 2020). Moreover, COOH-modified polystyrene particles (0.5 um) exposure for 24 h also induced cytotoxicity in intestinal, placental and embryonic cells (Helser et al., 2019). Finally, 20 um polypropylene particles reduced cell viability after 48 h exposure in human dermal fibroblast (HDF) (Hwang et al., 2019). Cellular uptake of microplastics was observed in 3 studies under different conditions (Table 5). (Helser et al., 2019; Stock et al., 2019; Wu et al., 2019). Stock et al. (2019) showed that 4 um polystyrene particles were preferentially internalized and among all the cell types tested (mucus co-culture, M model and Caco-2 cells), macrophages had the highest ability to internalize the particles. Additionally, prolonged exposures resulted in an increase in intracellular particles. Moreover, COOH-modified polystyrene of 0.5 um were also observed intracellularly in intestinal and placental cells (Helser et al., 2019). Fluorescent polystyrene (0.1 um) particles were found colocalized with lysosomes in Caco-2 cells (Wu et al., 2019).

One study (Wu et al., 2019) showed that polystyrene particles might be exerting their toxicity through ABC transporters. 0.1 um size polystyrene particles greatly inhibited ABC transporters in Caco-2 cells (Wu et al., 2019) and larger (5 um) polystyrene particles were only able to inhibit the transporter at higher concentrations. Moreover, co-exposure of microplastics and arsenic showed that the intracellular concentration of arsenic in cells exposed to arsenic-coated polystyrene increased compared to the arsenic-only exposure. Additionally, when artificial ABC inhibitors were added, 0.1 um particles accumulated intracellularly. Therefore, the authors suggested that 0.1 um size polystyrene particles might exacerbate other contaminant-induced toxicity by acting as substrates of ABC transporters and reducing the transport capacity of other substrates. However, since 5 um particles did not act as a substrate, they suggested that they could inhibit ABC transporter activity by mitochondrial depolarization and subsequent depletion of ATP (Table 5).

Oxidative stress was exacerbated at different experimental conditions in 5 studies (Schirinzi et al., 2017; Hwang et al., 2019; Wu et al., 2019; Dong et al., 2020; Wu et al., 2020) (Table 6). Direct measurement of ROS showed an increase after exposure to polystyrene (0.1 and 0.5 um) for 12 h, polystyrene (10 um) for 24 h, polyethylene (3–16 um) for 24 h and polypropylene for 6 h (20 um when administered in DMSO). Polystyrene particles increased heme oxygenase-1 (HO-1) enzyme levels, which is directly involved in oxidative degradation (Dong et al., 2020), and inhibited catalase activity (Wu et al., 2020). Additionally, polystyrene-arsenic co-exposure increased ROS levels compared to arsenic-only exposure (Wu et al., 2019).

Membrane integrity was compromised after 12 and 24 h polystyrene exposure in two studies (Wu et al., 2019 and Dong et al., 2020) (Table 6). Polystyrene exposure also induced mitochondrial membrane depolarization (Wu et al., 2019), TEER value decrease (Dong et al., 2020), Zonula Occludens-1 (ZO-1) expression decrease (Dong et al., 2020) and ATT (Dong et al., 2020) level increase, further suggesting membrane destabilization. However, COOH-modified polystyrene particles did not cause any effect in the cellular membranes of the gastrointestinal tract (GIT) or placental barrier co-culture models (Hesler et al., 2019).

Immune response was assessed by four studies and results greatly varied depending on the experimental conditions (Table 6). 24 h exposure to polystyrene particles of 5 um size upregulated 4 inflammation genes (Wu et al., 2020) and 1.72 ± 0.26 um particle size polystyrene increased IL-6 and IL-8 levels (Dong et al., 2020). However, small size (20 um) polypropylene particles only increased IL-6 levels at high concentrations (100–1,000 ug/ml) and TNF-α increased after 100 ug/ml after 20 um size exposure in peripheral blood mononuclear (PBMC) cells (Hwang et al., 2019). Polypropylene particles (20 um, 25–200 um) induced histamine release in mast (HMC-1) cells (Hwang et al., 2019). However, polystyrene particles exposure for 24 and 72 h time points did not induce differentiation of macrophages (Stock et al., 2019).

Other endpoints, such as, genotoxicity, gene expression, embryotoxicity and hemolysis, were also investigated to a lesser degree in some of these studies. Hwang et al. (2019) showed that polypropylene particles of 20 um and 25–200 um sizes can induce hemolysis in sheep red blood cells. Hesler et al. (2019) showed no genotoxic potential of COOH-modified polystyrene particles (0.5 um) by a p53 reporter assay in HepG2CDKN1A-DsRed and micronucleus assay in CHO-KI cells after 24 h exposure. Moreover, their study also showed that 0.5 um polystyrene are weakly embryotoxic after 24 h exposure.

Wu et al. (2020) carried out expression analysis on Caco-2 cells exposed for 24 h or 48 h to 5 um polystyrene particles at concentrations of 12.5 or 50 mg/L. RNA-Seq analysis after 24 h showed 80 upregulated differentially expressed genes (DEGs) and 94 downregulated genes. The GO terms after 12.5 mg/ml compared to 50 mg/ml shows a shift from enriched metabolism pathways to cancer pathways, which was consistently observed after 48 h exposure, where 210 DEGs were observed. RT-PCR on cells exposed for 24 h to polystyrene showed five proliferation related genes (Ras, ERK, MER, CDK4, Cyclin 1D) were downregulated and four inflammation related genes (TRPV1, iNOS, IL-1β, IL-8) were up-regulated.

#### Workers in Plastic Factories Develop Dermatoses

Search results showed 10 epidemiological studies that investigated the effect of different toxicants involved in plastic manufacturing and associated risk of developing pathologies. Those studies analyzed health records from workers occupationally exposed to nylon fibers (Kern et al., 1998), poly(vinyl acetate) fibers (Morinaga et al., 1999), epoxy resin (Jolanki et al., 1996), fiberglass reinforced plastic (Minamoto et al., 2002a and Minamoto et al., 2002b), acrylonitrile (Felter et al., 1997; Wood et al., 1998), glycidyl ether (Lanes et al., 1994), styrene (Sass-Kortsak et al., 1995) and glycerol polyclycidyl ether (Watkins et al., 2001).

Four studies showed that not only chemical additives but also plastic dust causes mechanical and contact dermatitis in workers. Briefly, Kern et al. (1998) studied 165 workers from a nylon flocking industry and showed increased risk interstitial lung diseases. Moreover, from 150 workers of a ski factory occupationally exposed to epoxy resin, 22 developed skin diseases such as allergic contact dermatitis (Jolanki et al., 1996). From 149 workers of fiberglass-reinforced plastics factories studied for skin diseases, 22 developed skin dermatoses, 7 were diagnosed with allergic contact dermatitis due to exposure to chemical, 3 developed irritant contact dermatitis and interestingly, 3 developed dermatitis due to mechanical irritation from glass fibers or dust and 9 developed allergic contact dermatitis and/or mechanical irritation dermatitis. Diagnosis was carried by sticking patches to the workers (Minamoto et al., 2002a). Minamoto et al. (2002b) further investigated the increased risk of developing skin diseases in workers from fiberglass-reinforced plastics. From 148 workers of fiberglass reinforced plastics factories studied, 87 (58.8%) developed skin problems.

The other 6 studies from our search showed no clear associations between the selected parameters (Lanes et al., 1994; Sass-Kortsak et al., 1995; Felter et al., 1997; Wood et al., 1998; Morinaga et al., 1999; Watkins et al., 2001).

## DISCUSSION

This review provides an opportunity to look at different fields of research that work towards the same objective, isolation and characterization of microplastic exposure and the identification of toxicological effects in different species and models, allowing us to identify the data gaps and weaknesses. Moreover, this review aimed to include wildlife species relevant to humans by using the One Environmental Health approach (Perez and Wise, 2018). Sea turtles and marine mammals include long-lived species that share a great variety with food sources and habitat with humans. While chronic exposures of over 10 years seem unfeasible under laboratory conditions, sampling these two groups of species provide insightful data on whole life exposures. However, a limitation that we faced on this review is the term microplastic is practically new, first used by Thompson et al. (2004), and therefore previous literature observing small size plastic debris could not be included.

Most observed data on microplastics exposure in sea turtles, marine mammals and humans concern ingestion. Nevertheless, as shown in human epidemiological studies, inhalation and dermal contact exposure are important routes of exposure that are overlooked by literature to date, forming a knowledge gap in the field. From the studies available, however, we identified 5 key parameters that any studies investigating microplastic exposure, no matter which route, should consider reporting: 1) concentration of microplastics, 2) average size, 3) shape, 4) color and 5) polymer type.

The concentration of microplastics found in each sample highly depends on the method of isolation. Overall, comparing the amount of microplastics per individual, marine mammal GI tracts contained more microparticles per individual. Specifically, the levels reported by Lusher et al. (2018) in small odontocetes from Atlantic Ocean were extraordinarily high for their body size. Scats from pinnipeds in the Pacific Ocean and fecal samples from human volunteers showed levels comparable to those found in the GI tract of odontocetes (Figure 1). Sea turtles overall contained lower levels of microplastics in the GI tract. The average size of the microplastics were between 0.1 and 5 um, however, more studies reported average sizes at the lower end of the range.

With respect to shape, GI tracts from sea turtles and marine mammals contained more fibers than fragments, while pinniped scat and human feces showed a higher proportion of fragments. These findings might suggest fibers have a longer residence time in the intestine. Fibers could be retained in the gut papillae due to shape plasticity and, therefore, might have a higher potential of toxicological effect due to a longer residence time in the gut. Such possibilities remain to be tested.

Blue, black, green and white/clear plastics are preferentially found in sea turtles and marine mammals, which is consistent with observations that they are the most frequent colors of microfibers in marine sediments (Gago et al., 2018). These same colors were found in pinniped feces along with red, purple, brown and yellow. Whereas, black and blue color plastic might be highly ingested due to their ubiquity in fishing gear, white/clear plastic has been hypothesized to be ingested by marine fauna because they mimic prey such as jellyfish (da Silva Mendes et al., 2015). Studies considering microplastics in humans did not report the color.

The polymer profiles found in sea turtles, marine mammals and human samples are similar. Polyethylene, polypropylene, ethylene propylene, polystyrene and polyester are found at high percentages in GI and fecal samples. However, other polymers such as poly(ethylene:prolypene:diene) rubber, polyamide (nylon), polyacrylamide, synthetic cellulose, polyoxymethylene, polycarbonate, polyvinyl chloride, polyurethane, polyvinyl ether, polybutylene terephthalate and polyether sulfone were also frequently observed. Those results correlate with the composition of microfibers and microplastics found in marine sediment and water column (Gago et al., 2018; Guo and Wang, 2019; Ajith et al., 2020). Among all, polyethylene and polypropylene are commonly found floating in the water column due to their low densities, which makes them more available for wildlife to ingest (Guo and Wang, 2019). It should not come as surprise the ubiquity of polyethylene polymer since it is extensively used in fishing gear (Chen et al., 2018) as well as packing food, plastic bags and bottles, among others. Interestingly, a review by Koelmans et al. (2019) showed that polyethylene, polypropylene, polyvinyl chloride, polyester and polystyrene are the most abundant polymers in drinking water. These outcomes suggest sea turtles, marine mammals and humans are being exposed to the same polymer types.

Microplastics are able to carry pollutants such as metals and organic pollutants through sorption, due to their distinct properties. Levels of organic pollutants have been measured across the globe indicating that PAH levels in microplastics are of special concern in East Asia and South America (Guo and Wang, 2019). However, in comparison fewer studies considered metals in microplastics from the marine environment (Guo and Wang, 2019), which is of special concern since laboratory and field studies have demonstrated that microplastics act as vectors for metals. Moreover, whole digestive system *in vitro* method (WDSM) have shown changes in bioavailability of metals adsorbed onto microplastics such as Cr(VI) through the digestive process and changes in shape and size of the particles.

Toxicity data on marine plastics in sea turtles, marine mammals and humans are limited. Cell culture studies indicate microplastics may cause cytotoxicity, oxidative stress, intracellular uptake, produce immune response, induce changes in the membrane, alter gene expression, cause weak embryotoxicity and hemolysis. Notably, most studies treated the cells only for acute (24 h) exposures, whereas for the investigation of effects of microplastics in the gut, a more prolonged exposure is more relevant. In humans a normal transit time is between 24 h and 48 h, or even 96 h depending on the diet (de Vries et al., 2015). The gut passage time and excretion time of microplastics in marine mammal species are largely unknown, and likely depend on the anatomical features of the GI tract of each species, the diet and the type of the plastic ingested. However, gut passage time in seals was calculated to be around 6 days (Grellier et al., 2006), whereas in turtles the ingesta passage time was 23 days. Therefore, data from prolonged exposures are essential. Additionally, unified reporting of units is also needed. As suggested by Karami (2017) in a review on the gaps in aquatic toxicological studies of microplastics, and as routinely used in particles toxicology, the best units to report concentration of microplastic in laboratory-based experiments is weight per unit of the surface area (example g/cm^2^).

In this review, variability in data collection made it challenging to compare number of microplastics, size and polymer types between studies. More standardization of sample preparation, digestion and isolation, characterization and quality control procedures will be key for the field to advance and allow more consistent reporting of data to allow for clearer comparisons. For example, in the wildlife research we found that, an important factor to take into consideration is the percentage of sample analyzed. Not analyzing the whole GI tract introduces variability in the results, since the occurrence of microplastics across the gut is not homogeneous (Lusher et al., 2015; Nelms et al., 2019). Digestion is another critical step in microplastic isolation and can lead to their destruction. Other sources of biases are mesh or filter sizes and the techniques used for polymer identification such as FITR, which directly influence the types of microplastics that are detected. Additionally, we found that using blank controls through the sample preparation, microplastic identification and characterization steps is necessary to account for external contamination.

## CONCLUSIONS

The characterization of physico-chemical properties of microplastics in sea turtles, marine mammals and human have shown that both wildlife and humans are likely being exposed to the same microplastics profiles. This conclusion is consistent with these three groups having similar major routes of exposure; inhalation, dermal contact and ingestion. From the available literature, we found that the five key parameters mentioned above: concentration, average size, shape, color and polymer type seem to be similar across the literature reviewed here.

Most of the studies regarding microplastics study the presence and characterization of microplastics in the GI tract and fecal samples. However, although ingestion in a major route of exposure, dermal and inhalation exposures are also a health concern. Epidemiological studies have linked exposure to toxicants involved in plastic manufacturing, such as additives and fiber dust, with contact and mechanical dermatitis and fibers localized in lung tissues have been suggested to increase risk of lung disease. However, those routes of exposure are largely unexplored in humans as well as marine mammals and sea turtles, indicating a significant knowledge gap in the field. Although data on human exposure to microplastics is currently limited, this field is rapidly developing and it is expected that in the future, new datasets and methodologies might allow for a better understanding of the exposure.

Additionally, even if the full toxicological profile of microplastics is largely unknown due to their complexity, *in vitro* studies have shown the ability of microplastics to induce immune response, oxidative stress, cytotoxicity, alter membrane integrity and cause differential expression of genes. However, these studies only investigated exposure to polystyrene, polyethylene and polypropylene polymer type microplastics and short-term exposures. Due to physiological relevance, more effort on prolonged exposures is needed.

## Figures and Tables

**TABLE 1 |:** Microplastics presence in the gastrointestinal tract of 7 species of sea turtles.

Location	Species	*n*	No of Microplastics	Size	Shape	Color	Polymer type	Ref.

Pacific Ocean (Cairns)	*Chelonia mydas*	2	3.5 microplastics/turtle	Particles in juvenile ranged from 0.45 to 2.51 mm. Particle in the adult female ranged from 0.76 to 2.95 mm	Particles and a microfilm (in adult female)	Out of 7 items, 3 clear particles, 1 clear film, 1 dark green particle, 1 black particle and 1 white particle	EAA, PVA, a particle composed of cotton: Olefin: PES and one mixed yarn synthetic fabriccomposed of cotton: Olefin: PES and one mixed yarn synthetic fabric	Caron et al. (2018)
Pacific Ocean (Queensland)	*Chelonia mydas*	7	11 particles/turtle^a^	Average fiber size 2.85 ± 0.23 mm.	Fibres 64.8%, fragments 20.2% and microbead 4.8%	Blue 44.9%, black 39.1%, red 8.6% and clear 2.9%	Elastomers 3.4% (e.g., EPDM rubber), synthetic regenerated synthetic regenerated CL fibres 68.9%, PE, EP, PET, PAM 27.7%	Duncan et al. (2019)
	*Caretta caretta*	3	6 particles/turtle^a^	Fragment and bead average diameter 0.26 ± 0.01 mm				
	*Natator depressus*	4	6 particles/turtle^a^					
	*Eretmochelys imbacata*	1	5 particles/turtle^a^					
	*Lepidochelys olivacea*	1	4 particles/turtle^a^					
Mediterranean Sea (Northern Cyprus)	*Chelonia mydas*	34	10 particles/turtle^a^	Average fiber size 1.40 ± 0.54 mm (mean ± S.E). Fragment and bead average diameter 0.07 ± 0.01 mm	Fibres 85.3%, Fragments 14.7%	Blue 34.4%, black 31.3%, red 18.2% and clear 9.9%	Elastomers 61.2% (e.g., EPDM rubber), woven synthetics 4.9%, synthetic regenerated CL fibres 5.8% and PE, EP, PET, PAM (total 20.7%)	Duncan et al. (2019)
	*Caretta caretta*	22	12.5 particles/turtle^a^					
Atlantic Ocean (North Carolina)	*Chelonia mydas*	10	5 particles/turtle^a^	Average fiber size 2.87 ± 0.20 mm.	Fibres 77.1% Fragments 22.9%	Blue 36.3%, black 43.7%, red 17.5% and clear 2.5%	Synthetic regenerated CL fibres 63.2%, PE, EP, PET and PAM (total 36.8%)	Duncan et al. (2019)
	*Caretta caretta*	8	2.5 particles/turtle^a^	Fragment and bead average diameter 0.31 ± 0.04 mm				
	*Lepidochelys kempii*	10	3 particles/turtle^a^					
	*Dermochelys coriacea*	2	4 particles/turtle^a^					
Atlantic Ocean (Azores islands)	*Caretta caretta*	24	95 microplastics total in 58% of the turtles. 3.95 items/turtle	1–5 mm	Fragments (87%), sheets (8%) and pellets (5%)	Blue, green, and white most predominant	PE (60%), PP (20%) and different polymer mixtures (12%)^^^	Pham et al. (2017)
Atlantic Ocean and Indian Ocean (Sourthern Cape)	*Caretta caretta*	16	Fragments 12.2 ± 14.9/turtle (range 0–50). Pellets 0.6 ± 1.3/turtle (range 0–5). In total 229 fragment and 10 pellets	Average fragment 3.0 ± 1.4 × 1.0 ± 0.5 mm and average pellet size 3.9 ± 0.5 × 3.4 ± 0.8 × 1.4 ± 0.7 mm	Among all items: Fragments 76%. Pellets 3%	Fragments mostly white/cream, clear or blue/purple and pellets mostly black/grey/brown, white/cream and clear	NA	Ryan et al. (2016)

a Extrapolated from Figure 2 in Duncan et al., 2019;

^ Authors took into account all the items (macro, meso and microplastics) found; Abbreviations: polyethylene acrylic acid EAA, polyvinyl acrylic PVA Polyethylene PE, Ethylene propylene EP, Polyester PES, Polyacrylamide PAM, Polypropylene PP, Polystyrene PS, Polyamide (nylon) PA, Cellulose CL.

**TABLE 2 |:** Microplastic Presence in the Gastrointestinal Tract of 15 Cetacean and two Pinniped Species (in grey).

Location	Species	n	Microplastics/ Individual	Size	Shape	Color	Polymer type	References

Pacific Ocean (China)	*Neophocaena asiaeorientalis sunameri*	7	19.1 ± 7.2	NA	Fibers (70.1%). Sheets (14.9%), fragments (13.4%), and foam (1.5%)	Most were blue. Red, transparent, yellow, green	Most abundant: PP. Others found: PE, PA, PS, PC, and PET	Xiong et al. (2018)
	*Sousa chinensis*	3	2 adults (total 30 and 45)^a^ and the calf (2)	Average size 2.2 mm ± 0.4 (0.1 to 4.8 mm)	Fibers (70.3%). Fragments and flakes were also found	Most were white and blue	Most abundant: PES, Others found: PP, CL, PE, PA and PBT.	Zhu et al. (2019)
Arctic Ocean (Canada)	*Delphinapterus leucas*	7	11.6 ± 6.6 (total 97 ± 47)^a^	Size range 0–5 mm >1 mm most abundant	Fibers (49%) and fragments (51%)	NA	Most abundant: 44% PET (85% fibers). Others found: PVC, PO, PA, acrylic, PP, PS, PE.	Moore et al. (2020)
Atlantic Ocean (Netherland, Spain, Ireland, Scotland, England, Wales)	*Megaptera novaeangliae*	1	6 (total 167)^a^	Average size 1.1–4.7 mm by 0.4–2.4 mm	Sheets and fragments were found. Fibers not counted due to lack of blanks	NA	Most abundant: PE, PA. Others found: PP, PVC and PET.	Besseling et al. (2015)
	*Delphinus delphis*	35	12 ± 8 (range 3 to 41)	Fibers 2.11 ± 1.26 mm. Fragments 1.29 ± 0.93 mm. Beads 0.95 mm	Fibers (96.59%), fragments (3.16%), beads (0.24%)	Blue (45.26%), black (24.57%), green (15.58%), red (14.36%)	NA	Hernandez-Gonzalez., et al. (2018)
	*Ziphius cavirostris*	1	53	Most abundant sizes 1 to 5 mm. Size range 0.3 to 16.7 mm	Fibers (83.6%) and fragments (16.4%)	Blue (29.2%), grey (18.2%), black (16.8%) and orange (15.05%)	NA	Lusher et al. (2018)
	*Delphinus delphis*	9	36.25 ± 19.36^b^					
		(4)^+^						
	*Stenella coeruleoalba*	2	44.5 ± 16.26^b^					
	*Phocoena*	5	33 ± 23.07^b^					
	*phocoena*	(3)^+^						
	*Orcinus orca*	1	39					
	*Tursiops truncatus*	2	35 ± 21.92^b^					
		(1)^+^						
	*Mesoplodon mirus*	1	88	Mean length 2.16 mm ± 1.39 (0.3 to 7 mm)	Most were fibers and fragments. Film was also found	NA	Most abundant: Rayon (53%) Others found: PET, acrylic, PP, PE.	Lusher et al. (2015)
	*Phocoena phocoena*	21	5.23 ± 2.53^b^	Average fiber size 2 mm ± 2.3 mm (2 cm to 0.1 mm). Average fragments size 0.9 mm ± 1.1 (4 × 2 mm to 100 × 100 um)	Fibers (84%) and fragments (16%)	Most were blue (42.5%), black (26.4%), clear (12.8%)	Most abundant: Nylon 60%. Others found: PE, PET, PES, phenoxy resin, PE, PP and rayon, PA and LDPE.	Nelms et al. (2019)
	*Stenella coeruleoalba*	1	7					
	*Tursiops truncatus*	1	6					
	*Delphinus delphis*	16	5.69 ± 3.34^b^					
	*Grampus griseus*	1	9					
	*Kogia breviceps*	1	4					
	*Lagenorhynchus albirostris*	1	3					
	*Lagenorhynchus acutus*	1	8					
	*Phoca vitulina*	4	4.25 ± 2.5^b^					
	*Halichoerus*	3	6 ± 2^b^					
	*Halichoerus grypus*	13	27.9 ± 14.7	NA	Fibers (86%), fragments (14%) and films (1%)	NA	NA	Hernandez-Millan et al. (2019)

a estimated from the analysis of a section. Abbreviations: Polyethylene PE, Low density polyethylene LDPE, Ethylene propylene EP, Polyester PET, Polyacrylamide PAM, Polypropylene PP, Polystyrene PS, Polyamide (nylon) PA, Polycarbonate PC, Polybutylene terephthalate PBT, Polyvinyl chloride PVC, Polyether sulfone PES, Cellulose CL, Polyolefin PO.

b average calculated from supplementary data; + number individual where intestines were analyzed.

**TABLE 3 |:** Microplastic content in feces from 8 pinniped species and 1 odontocete species (in grey).

Location	Species	n	No of microplastics	Size	Shape	Color	Polymer type	Ref

Atlantic Ocean (Cornish Seal sanctuary and Massachusetts)	*Halichoerus Grypus* ^^^	31 from 4 resident seals	48% of scats contained microplastics. Ranging 0 to 4 particles/scat.^a^	Fragments size from 0.4 × 0.3 mm to 5.5 × 0.4 mm. Fibers from 0.6 to 3.5 mm	Fragments (69%) and fibers (31%)	Black (27%), transparent and red (23% both), blue (15%), and orange (12%)	EP (27%), PP (27%), PE (12%). Other polymers were also found	Nelms et al.(2018)
	*Phoca vitulina vitulina*	32	2 fragments in 32 scats	Size ranged 1.2 to 3.5 mm	Fragments	Tan, red, purple and white	Alkyd resin (1), celophane (2), EPDM rubber(1)	Hudak et al.(2019)
	*Halichoerus grypus atlantica*	129	2 fragments in 129 scats					
Pacific Ocean (Australia, Alaska, California, Peru, Chile)	*Arctocephalus tropicalis Arctocephalus gazella*	145	164 plastic items in total. Mean 1.13 particle/scat*	Mean length 4.1 mm. Mean width 1.9 mm. Range 2 mm to 5 mm	Most were fragments with irregular shapes	White, brown, blue green and yellow were most common	PE 93%, PP 4% Other polymers were found	Erisksson and Burton (2003)
	*Callorhinus ursinus*	44	398 fragments and 186 fibers in total. 9.05 fragments/scat and 4.22 fibers/scat	82% of microplastics below 1 mm and 72% fibers below 2 mm	Fragments and fibers. Fibers were also present in the laboratory blanks and sediment samples	Fragments were white. Fibers were black, white, purple, blue, red, yellow and clear	Fragments were low density PE. Only two fragments tested and fibers were NA.	Donohue et al.(2019)
	*Arctocephalus australis*	50	8.84 ± 11.01 fibers/scat and 1.5 ± 5.78 fragments/scat*	NA	Fibers more abundant. Fragments were also present	Most abundant. color was blue and white	81.5% of fragments or fibers were anthropogenic in origin. 51.5% were cotton and 30% were polymers (PET and PA), the rest did not match any spectra	Perez-Venegas et al. (2020)
	*Arctocephalus philippii*	40	29.75 ± 49.1 fibers/scat and 1.5 ± 6.36 fragments/scat*					
	*Otaria byronia*	14	75.57 ± 81.46 fibers/scat and 1.28 ± 4.8 fragments/scat*					
		12	23.08 ± 16.18 fibers/scat and 1.25 ± 3.1 fragments/scat*					
		10	29.2 ± 26 fibers/ scat and 0.4 ± 1.26 fragments/scat*					
	*Arctocephalus australis*	79	23.97 ± 34 fibers/scat and 0.16 ± 1.46 fragments/scat*					
	*Arctocephalus australis*	51	Microfibers in 67% of examined samples. Ranging from 0 to 180/scat	>0.1 mm	Microfibers	Blue (45%), white (24%), black(16%), red (15%)	NA	Perez-Venegas et al. (2018)
Arctic Ocean (Canada)	*Delphinapterus leucas* ^a^	2	2 and 0 items	Range was 0–5 mm. Most were <1 mm	Fragments (51%) and fibres (49%)	NA	Most abundant 44% PES (85% fibres). Others: PVC, PO, PA, acrylic, PP, PS, PE.	Moore et al.(2020)

* calculated from data in the paper.

^ Seals from Cornish Seal Sanctuary, UK ^a^ results are outcomes from GI tract and feces content

a subsample analyzed. Abbreviations: Polyethylene PE, Ethylene propylene EP, Polyester PET, Polypropylene PP, Polystyrene PS, Polyamide (nylon) PA, Polyvinyl chloride PVC, poly(ethylene:prolypene:diene) EPDM, Polyolefin PO.

**TABLE 4 |:** Presence of microplastics in human samples: Feces and lungs.

Endpoint	n	Method	No of microplastics	Size and shape	Polymer type	Ref

Microplastics isolation from human stool	8	*Chemical digestion of organic material. *Filtration through a 50 um metal sieve. *Resuspended in ultrapure water, filtered via vacuum system and dried.*Polymer composition by FTIR	100% samples had microplastic. Median: 20 microplastics/10 g (range 18 to 172)	Size range from 50 to 500 um sizes. Most were fragments or films. Rarely spheres and fibers	9 types: PP, PET, PS, PE, POM, PC, PA, PVC, PU The most abundant PP and PET (present in all samples)	Schwabl et al.,(2019)
	10	*Fenton’s reagent and nitric acid digestion* vacumm filtration steps in between digestions* polymer composition by Raman spectra	40% samples had microplastic	>1 um	The microplastics were identified as PBT and PVB particles	Yan et al.,(2020)
Presence of plastic fibers in human lung tissue	114	*Fresh lung specimens were analyzed in dual-slide chambers under white light, fluorescent light, polarizing light and phase contrast light. *Paraffin embedded lung tissue histopathological slides were analyzed	87% samples had fibers. 83% of nonneoplastic lung specimens and 97% of malignant lung specimens contained inhaled fibers		The histopathological slides confirmed the presence of cellulosic and plastic fibers in the lungs identified by polarized light	Pauly et al.,(1998)

Abbreviations: Polyethylene PE, Polyester PET, Polypropylene PP, Polystyrene PS, Polyamide (nylon) PA, Polyvinyl chloride PVC, Polyoxymethylene POM, Polybutylene terephthalate PBT, Polyvinyl ether PVE, Polycarbonate PC, Polyurethane PU.

**TABLE 5 |:** Effects of microplastics in cell viability and uptake.

End-point	Polymer type	Exposure time	Size	Concentration range	Assay	Cell type	Outcome	Ref

Cell viability	Polystyrene (PS)	12 h	0.1 and 5 um	1–200 ug/ml	CCK-8 kit	Caco-2 cells	No effect was observed	Wu et al.(2019)
		24 h, 48 h	1.72 ± 0.26 um	1–1,000 ug/cm^2^	Trypan blue	BEAS-2B	Viability decreased to 60–70% at 1,000 ug/cm2 after 24 h exposure and all the concentrations above 10ug/cm2 after 48 h exposure	Dong et al. (2020)
			1, 4 and 10 um	1–1,000 ug/ml	CTB and MTT	Caco-2 cells	CTB: 24 h 48 h exposures decrease the viability to 0% only after 1um PS exposure. MTT: Showed the same, results and additionally cell viability decreased to 80% and 70% after 24h and 48 h exposure to 4um	Stock et al. (2019)
			5 um	0.00001–100 ug/ml	MTT	Caco-2 cells	No effect was observed	Wu et al. (2020),
		24 h	10 um	0.05–10 mg/L	Hoechst 33258	T98G and HeLa cells	No effect was observed	Schirinzi et al., (2017)
	COOH-modified polystyrene (PS)	24 h	0.5 um	0.01–100 ug/ml	WST-1	GIT co culture model	Intestinal cells: PS decreased the metabolic activity only at 0.01 μg/ml. Placental cells: PS increased mitochondrial activity only at concentrations from 0.01–10 μg/ml	Helser et al.,(2019)
					MTS	BeWo b30 cells		
	Polyethylene (PE)	24 h	3–16 um	0.05–10 mg/L	Hoechst 33258	T98G and HeLa cells	No effect was observed	Schirinzi et al., (2017)
	Polypropylene (PP)	48 h	20 and 25–200 um	In DMSO 10–1000 ug/ml and in powder 0.1–4.5 mg	CCK-8 colorimetric kit	HDF	HDF cells: only the 20 um PP (in DMSO) caused a reduction in viability (20%) at the highest concentration 1000 ug/ml	Hwang et al., (2019)
Intracellular localization	Polystyrene (PS)	12 h	0.1 and 5 um	1–80 ug/ml	ABC transporter activity (CAM cell probe)	Caco-2 cells	Inhibition of ABC transporter was observed for 0.1 um PS concentrations >20 ug/ml and 5 um PS only at 80 ug/ml	Wu et al., (2019)
		24 h	1, 4 and 10 um	10^8^/ml (1 and 4 um), 3×10^6^/ml (10 um)	Fluorescence microscopy	Caco-2 cells, mucus co-culture^1^ model and M-cell model^2^	4 um PS were absorbed the most in Caco-2 cells (3.8%), M cell model and mucus model 4.8%). 1 um PS were significantlyabsorbed by the M cell mode (5.8%)*	Stock et al.,(2019)
		24 h, 72 h	1, 4 and 10 um	100,000/ml (1 um), 250,000/ml (4 um), 60,000/ml (10 um)	Fluorescence microscopy	THP-1 cells derived macrophages	Macrophages contained intracellular 4 um PS (40–80%) and 1 um and 10 um in lower extent	Stock et al., (2019)
	COOH-modified	24 h	0.5 um	100 ug/ml	Confocal microscopy	GIT barrier^3^ and placental barrier coculture^4^ models	In the GIT barrier coculture, PS were internalized by intestinal cells and in the placental barrier model the placental cells	Hesler et al., (2019)
	Fluorescent polystyrene (PS)	12 h	0.1 um and 5 um	20 ug/ml	Confocal microscopy	Caco-2 cells	Overlap between lysosomes and microplastics. Level of 5 um entering into cells lower than 1 um	Wu et al. (2019)
	Polystyrene (PS) and arsenic (As)	12 h	0.1 and 5 um	PS: 20 ug/ml (0.1 um), 80 ug/ml (0.5 um). As: 150 mg/L	Intracellular arsenic by ICP-MS	Caco-2 cells	0.1 um PSs at 20 ug/ml increased the intracellular concentration of As	Wu et al. (2019)

* extrapolated from the graph. Abbreviations: Polystyrene PS, Polyethylene PE, Polypropylene PP.

1 Caco-2 cells and HT29-MTX-E12 cells

2 Caco-2 and RajiB transwell coculture

3 Caco-2 and HT29-MTX-E12 cells

4 BeWo b30 and HPEC-A2 cells.

**TABLE 6 |:** Effects of microplastics in oxidative stress and membrane integrity.

End-point	Polymer type	Exposure time	Size	Concentration range	Assay	Cell type	Outcome	Ref

Oxidative stress	Polystyrene (PS) and arsenic (As)	12 h	0.1 and 5 um	20 ug/ml (0.1 um), 80 ug/ml (0.5 um) As:75 and 150 ug/l	DCFH-DA kit assay	Caco-2 cells	ROS increased after the co-exposure (PS + As), comparing to just arsenic exposure	Wu et al. (2019)
	Polystyrene (PS)	12 h	0.1 and 5 um	1–200 ug/ml	DCFH-DA kit assay	Caco-2 cells	ROS production only increased after 200 ug/ml exposure to 0.1 um and 5 um PS.	Wu et al. (2019)
		24 h	1.72 ± 0.26 um	10–1000 ug/cm^2^	Western blot (HO-1) DCFH-DA kit assay	BEAS-2B cells	HO-1 protein level significantly increased after 10 and 1000 ug/cm^2^ DCFH-DA increased at 1000 ug/cm^2^	Dong et al. (2020)
			10 um	0.05–10 mg/L	DHE solution	T98G and HeLa cells	ROS increased in both cell lines. EC50 9.6 mg/L in T98G and EC50 13.56 mg/L in HeLa cells	Schirinz et al. (2017)
		24 h, 48 h	5 um	12.5–50 mg/L	SOD, GSH, MDA detection and CAT activity	Caco-2 cells	No effect on SOD, GSH and MDA levels. Activity of catalase was inhibited only after 24 and 48 h exposure to 50 mg/L of 5 um PS	Wu et al (2020).
	Polyethylene (PE)	24 h	3–16 um	0.05–10 mg/L	DHE solution	T98G and HeLa cells	ROS increased only in T98G cells. EC50 41.22 mg/L	Schirinzi et al. (2017)
	Polypropylene (PP)	6 h	20 and 25–200 um	50–1,000 ug/ml in powder and DMSO	DCFH-DA kit assay	HDF cells	ROS increased after exposure to 20 um PP (in DMSO) at 1000 ug/mL. When administered in powder, ROS did not increase	Hwan et al. (2019)
Membrane integrity	Polystyrene (PS)	12 h	0.1 and 5 um	1–80 ug/mL	JC1 assay, LDH assay and TMA-DPH	Caco-2 cells	Mitochondrial membrane depolarization occurred after 20 to 80 ug/mL for 0.1 um PS and after all the concentrations of 5 um. No effects on LDH leakage or polarization	Wu et al. (2019)
		24 h	1.72 ± 0.26 um	10–1000 ug/cm^2^	TEER, ELISA (ZO-1 and AAT) and Western blot (ZO-1)	BEAS-2B cells	TEER value decreased in the epithelial barrier after 10 and 1,000 ug/cm^2^ exposure. ZO-1 levels decreased after exposure to 10 and 1000 ug/cm^2^. AAT level decreased after exposure to 1,000 ug/cm^2^	Dong et al. (2020)
	COOH-modified polystyrene (PS)	24 h	5 um	10–100 μg/ml	TEER	GIT barrier and placental barrier coculture model	No effect in GIT or placental barrier were observed	Hesler et al. (2019)
Immune response	Polystyrene (PS)	24 h	1.72 ± 0.26 um	10–1000 ug/cm^2^	ELISA (IL-6, IL-8)	BEAS-2B cells	IL-6 significantly increased at 10 and 1000 ug/cm^2^ exposure. 1000 ug/cm^2^ exposure increased IL-8 expression	Dong et al. (2020)
		24 h, 48 h	5 um	12.5–50 mg/L	RT-PCR	Caco-2 cells	Four inflammation related genes (TRPV1, iNOS, IL-1β, IL-8) were up-regulated	Wu et al. (2020)
		24 h, 72 h	1, 4 and 10 um	100,000/ml (1 um), 250,000/ml (4 um), 60,000/ml (10 um)	Macrophage polarization (Western Blot and RT-PCR)	THP-1 cells	No macrophage differentiation	Stock et al. (2019)
	Polypropylene (PP)	4 h, 72 h, 4 days	20 and 25–200 um	10–1,000 ug/ml	ELISA (IL-2, IL-6, IL-10, TNF-α)	PBMC	PBMC: IL-6 increased after 20 um PP at 1,000 and 100 ug/ml and TNF-α increased after 100 ug/mL after 20 um size exposure	Hwang et al.,(2019)
		48 h	20 and 25–200 um	100 ug/ml and 500 ug/ml (20 um and 25–200 um)	Histamine by ELISA kit	HMC-1 cells	Histamine was released after exposure to 500 ug/ml 20 um PP in HMC-1 cells	Hwang et al.,(2019)

Abbreviations: PS, Polystyrene; PE, Polyethylene; PP, Polypropylene.
